# A dataset on vulnerabilities affecting dependencies in software package managers

**DOI:** 10.1016/j.dib.2025.111903

**Published:** 2025-07-21

**Authors:** A. Germán Márquez, Ángel Jesús Varela-Vaca, María Teresa Gómez López

**Affiliations:** i3US and University of Seville, Dept. of Computer Languages and Systems, IDEA Research Group, Seville, Spain

**Keywords:** Security, Vulnerability, PyPI, Package, RubyGems, Cargo, NPM

## Abstract

The increasing reliance on third-party dependencies in software development introduces significant security risk challenges. This study presents a dataset that maps the vulnerabilities that affect dependencies in three major package managers: Node Package Manager (NPM), Python Package Index (PyPI), Cargo Crates and RubyGems. The dataset comprises information on 4437,679 unique packages and 60,950,846 versions of packages, with vulnerability data sourced from Open Source Vulnerabilities (OSV). It includes 270,430 known vulnerabilities linked to package versions, allowing a detailed analysis of security risks in software supply chains. Our methodology involved extracting dependency and version data from official package manager sources, correlating them with vulnerability reports, and storing the results in structured formats, including CSV and database dumps. The resultant dataset enables automated monitoring of vulnerable dependencies, facilitating analysis and security assessments, and defining mitigation strategies. This work identifies that 0.42 % of PyPI, 7.5 % of RubyGems, 3.91 % of Cargo and 6.93 % NPM versions rely on at least one vulnerable dependency. Furthermore, PyPI has 329 latest versions affected, RubyGem 919, Cargo 53, and NPM 14,858. This dataset provides valuable information for researchers, developers, and security professionals looking to improve software supply chain security. It provides a foundation for developing tools aimed at security and data analytics, enabling early vulnerability detection and improving mitigation controls for dependency-related security risks, thus promoting more secure software ecosystems. The dataset can be extended by incorporating additional packages, introducing new features, and ensuring continuous updates.

Specifications Table

**Table:** 

Subject	Computer Sciences.
Specific subject area	Security for dependencies found in the Software Supply Chain for Software Development Projects.
Type of data	Raw data including csv files and database dumps, and analysed data including tables and figures.
Data collection	The data was extracted using HTTP calls to public APIs, or using publicly available database dumps from the original sources. The pipelines followed have been defined using Python code, and the data were indexed in our own databases; and finally, the resultant CSV analysed was generated from our own databases.
Data source location	Package data and versions were collected from the Node Package Manager (NPM), Python Package Index (PyPI), Cargo Crates and RubyGems package managers. Vulnerability data was collected from the Open Sources Vulnerabilities (OSV) allowing our dataset to have a larger number of vulnerability sources. These sources are: GitHub, PySEC, Go, Rust, Global Security Database, OSS-Fuzz, Rocky Linux, AlmaLinux, Haskell, RConsortium, OpenSSF, Python Software Foundation, Bitnami, and Ubuntu. In addition, the OSV team maintains a conversion pipeline that transforms Debian security advisories, Alpine SecDB and the National Vulnerability Database (NVD) into OSV format (for open source software).
Data accessibility	Repository name: Zenodo [1] Data identification number: 10.5281/zenodo.15432733 Direct URL to data: https://doi.org/10.5281/zenodo.15432733
Related research article	A. Germán Márquez, Ángel Jesús Varela-Vaca, María Teresa Gómez López, Jose Ángel Galindo, David Benavides, Vulnerability impact analysis in software project dependencies based on satisfiability modulo theories (SMT), Computers \& Security 139 (2024) 103,669. doi: https://doi.org/10.1016/j.cose.2023.103669. [2]

## 1 Value of the Data

• This dataset contains information related to 4437,679 software packages and 60,950,846 versions of the Node, Python, Rust, and Ruby package managers. Belonging to Node Package Manager (NPM) [3] 3461,263 packages and 50,943,372 versions, Python Package Index (PyPI) [4] 599,307 packages and 6875,330 versions, Cargo [5] 168,944 packages and 1393,371 versions, and RubyGems [6] 208,165 packages and 1738,773 versions.
• The dataset has been completed with 270,430 known vulnerabilities attached to any version of software packages. All of these have been extracted from the Open Source Vulnerabilities (OSV) [7].
• This dataset can help identify vulnerable dependencies (i.e., direct and indirect), enabling automated data analysis and monitoring of those that pose potential security risks to software development projects that use package managers.
• This dataset enables the diagnosis of which components need upgrading or replacement with more secure alternatives. It can assist in developing tools to facilitate the analysis and detection of vulnerabilities in third-party software packages.
• It can serve as a valuable resource in the state of the art for further studies in this field and for expanding the current dataset with additional data features, other package managers, and other vulnerability repositories.

## 2 Background

Today's software development projects delegate much of their functionality to third-party software components commonly referred to as dependencies. Due to the fact that core software depends on third-party components, they have become the cornerstone of the Software Supply Chain (SSC) [8].

Furthermore, securing the SSC remains an ongoing challenge, which requires continuous efforts to manage and maintain the security of software dependencies. For example, Sonatype has reported a staggering increase 650 % year over year in detected SSC attacks [9]. One key issue is the necessity of updating SSC packages, particularly when dealing with outdated dependencies or those containing known vulnerabilities [10]. Furthermore, another critical weakness lies in the lack of comprehensive information on vulnerable packages [11], which hinders proactive measures to mitigate risks. This visibility gap contributes to the success of high-profile supply chain attacks, such as those that affect SolarWinds, Log4j, and xz Utils, highlighting the urgent need for improved vulnerability tracking and security practices on using dependencies.

Previous research has examined malicious code found in packages inserted into PyPI [12,13] finding that many of them were mistakenly or intentionally affected. Other works such as Zerouali et al. [14] which analyse how packages in the RubyGems environment behave when vulnerabilities are discovered and when they are fixed; approximately 3 % of dependency vulnerabilities affecting projects and 40 % affecting packages have fixes available in newer releases within the same major version of the dependency. Although there exist security datasets, they focus on the analysis of package network traffic or protocols [15], or even to detect DDoS attacks on Internet of Things (IoT)-based network traffic [16]. To the best of our knowledge, there is no dataset that covers all packages indexed in the NPM, PyPI, Cargo, and RubyGems package managers while also including the known vulnerabilities affecting them.

## 3 Data Description

**Fig. 1:**
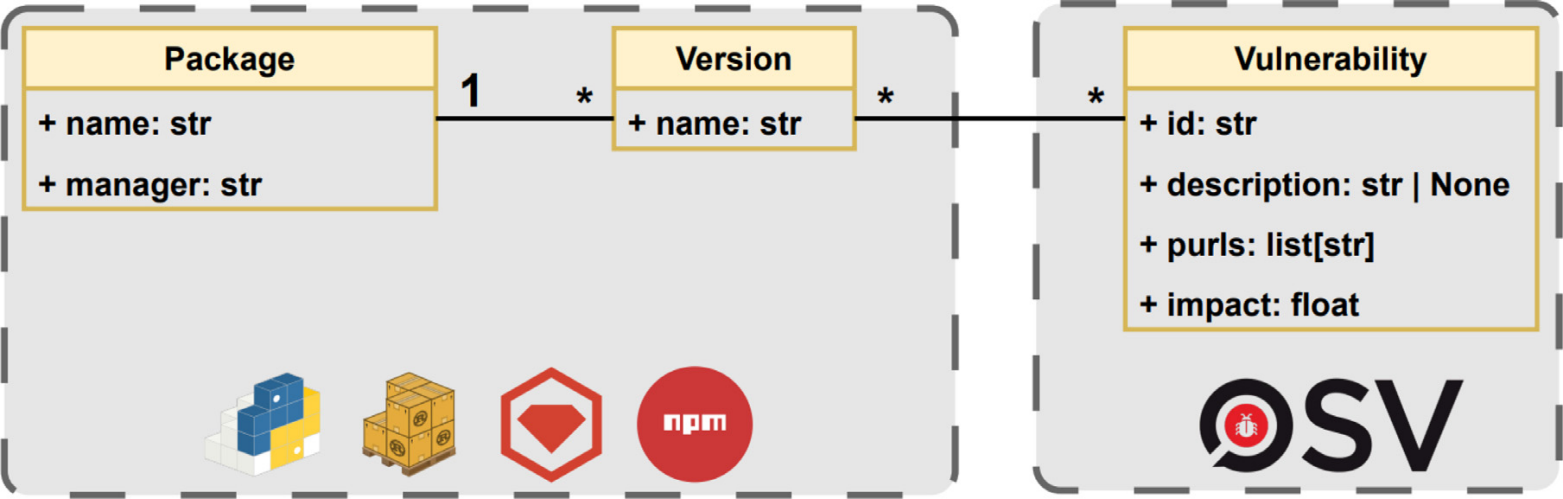
Data model.

Currently, package managers do not provide direct information on the vulnerabilities that affect their packages, with PyPI and NPM being the only one among those used in this work that does. For that reason, we have used OSV as the vulnerability database to feed our dataset. Fig. 1 shows the data model used to build the dataset. It consists of packages, versions, and vulnerabilities. The packages have a name and a manager (being the possibilities NPM, PyPI, Cargo, or RubyGems); it is associated with a set of versions with a name. For example, the package requests of PyPI and the set of versions ranging from *0.0.1* to *2.32.3*. Versions are associated with multiple vulnerabilities and vice versa. Each vulnerability contains its unique universal identifier, a description, affected products and severity (Common Vulnerability Scoring System (CVSS)). The affected products are composed by the name of the package, the ecosystem (package manager or operating system in which the package works) and the Package URL (purl) Specification.^1^ The purls have the following schema *pkg:type/namespace/name@version,* being the type of the ecosystem, the namespace is a prefix like the group in the Maven ecosystem, Docker Owner or GitHub organisation, the name and the version of the package. CVSS is a standardised method to qualitatively measure the severity of a security vulnerability with a numerical score ranging from 0 to 10. Consider factors such as impact on confidentiality, integrity, and availability, as well as attack complexity and required privileges. An example of three vulnerabilities is shown in Table 1. An example in the table is GHSA-222v-cx2c-q2f5 that is a Cross-Site Scripting (XSS) issue in phpMyAdmin versions before 5.2.2, allowing attackers to inject malicious scripts via crafted database or table names in the table maintenance feature. It has a CVSS v3.0 score of 3.5, which categorises it as a medium severity vulnerability.

**Table 1:** Example of vulnerabilities.

ID	Description	CVSS (Impact)	purls
GHSA-222v-cx2c-q2f5	An issue was discovered in phpMyAdmin 5.x before 5.2.2. An XSS vulnera…	**V3.x:** 6.4 MEDIUM	{pkg:composer/phpmyadmin/ phpmyadmin@5.2.1 ,…}
CVE-2024–21,907	Newtonsoft.Json before version 13.0.1 is affected by a mishandling …	**V3.x:** 7.5 HIGH	{pkg:nuget/Newtonsoft.Json@12.0.3, …}
PYSEC-2024–10	In Gentoo Portage before 3.0.47, there is missing PGP validation…	**V3.x:** 9.8 CRITICAL	{pkg:pypi/portage@3.0.46, …}

**Fig. 2:**
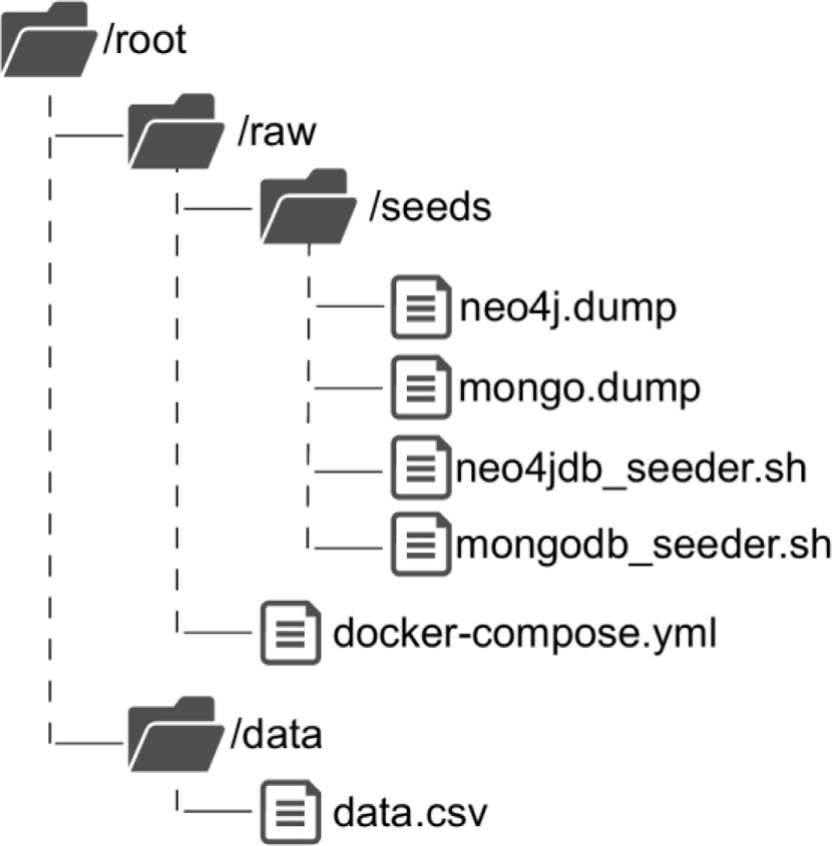
Folders structure.

Our project to build the dataset is organised as shown in Fig. 2. The root folder has two main folders, which are as follows:

• The first folder named *raw* contains raw data in *dump* format for packages and versions,and vulnerabilities. Using the *docker-compose.yml* file, these dump data files are loaded into two containers (using as sandboxes) in their respective databases using the execution of the script files: *graphdb_seeder.sh* and *vulndb_seeder.sh*. The attributed dependency graph is loaded into a Neo4J database, and the vulnerability information is loaded into a MongoDB database. MongoDB was chosen because vulnerabilities in the document format can be easily indexed and queried, while Neo4J is the most suitable database for managing, manipulating, and representing a graph.
• Data indexed in the dataset are also presented in *csv* format in the *data* folder. The *data.csv* aggregated all the information from both databases. The rows within the data.csv file follow the next structure for each vulnerability: *{package_name, package_manager, version_name, vuln_id, vuln_impact, vuln_description}*. In Table 2 are shown various example rows from the *csv* dataset.

**Table 2:** Example of rows within the dataset.

Package Name	Package Manager	Version	ID	CVSS (Impact)	Description
@angular/core	npm	6.0.0-rc.5	GHSA-c75v-2vq8–878f	5.4	A vulnerability was found in Angular…
nocodb	npm	0.91.1	GHSA-mx8q-jqwm-85mv	7.5	In NocoDB prior to 0.91.7,…
future	pypi	0.0.2	PYSEC-2022–42,991	0.0	An issue discovered in Python Charmers…
crossbeam-channel	cargo	0.4.3	GHSA-v5m7–53cv-f3hx	8.1	Impact the affected version of this crates…
addressable	rubygems	2.3.5	GHSA-jxhc-q857–3j6g	7.5	Impact within the URI template…

## 4 Experimental Design, Materials and Methods

**Fig. 3:**
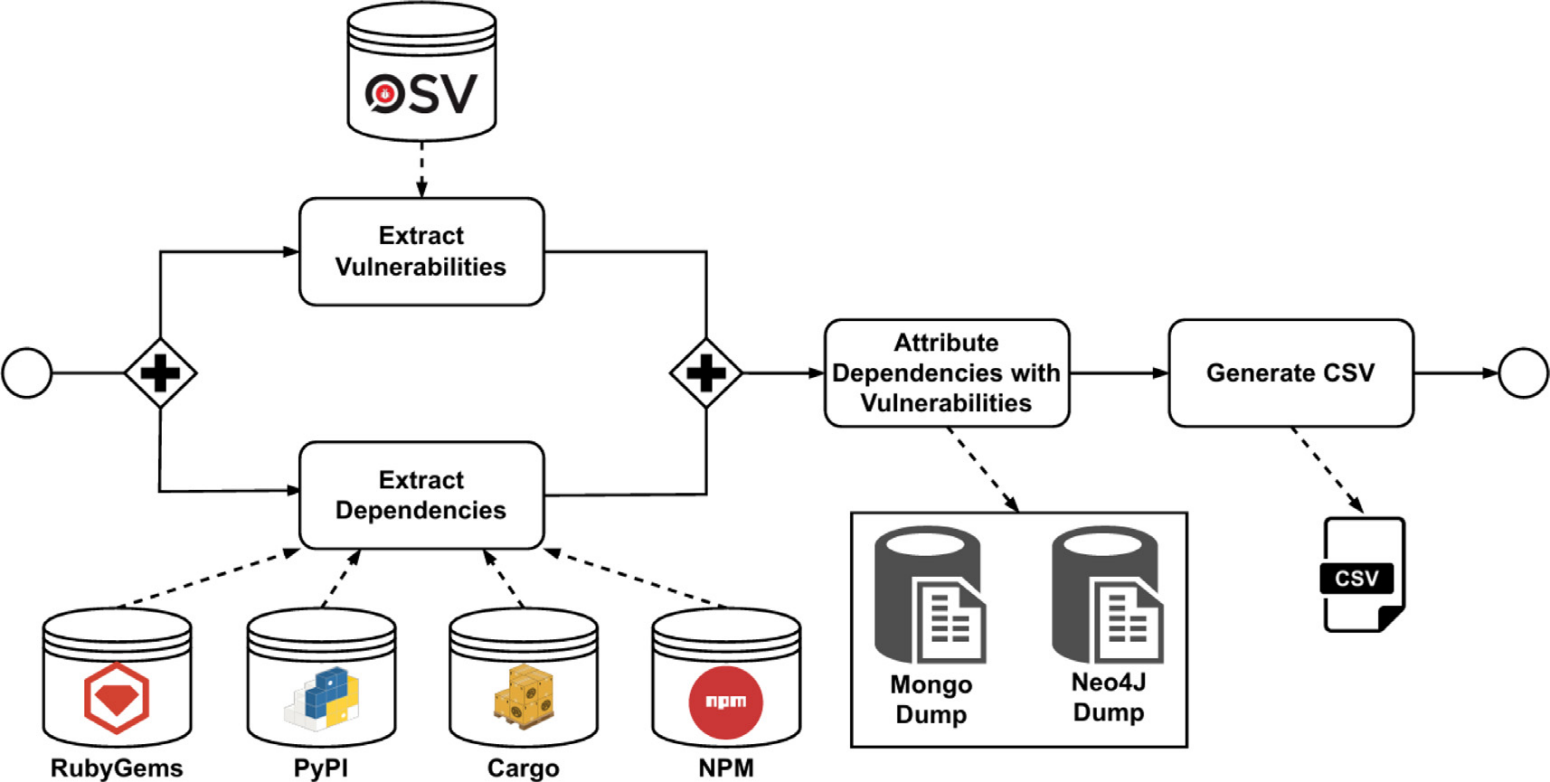
Dataset construction process.

Fig. 3 shows the process for the construction of the dataset. The process begins with two parallel tasks: the extraction of the dependencies of the package managers and the extraction of the vulnerabilities. The first task, extracting the name and versions of all packages included inside the package managers, was done using the NPM^2^ Replicate API, PyPI^3^ API, and the RubyGems^4^ and Cargo^5^ database PostgreSQL dumps provided in their official web pages. The second task, which extracts vulnerability information, was performed through the OSV API, from the vulnerabilities, the ID, the description, and the impact have been extracted.

After the extraction stage, the packages are attributed to the vulnerabilities extracted from the OSV using the purls associated with the vulnerabilities, using the properties *name* and *version* of the purl. For example, for vulnerability PYSEC-2023–132, which has the purl *pkg:pypi/copyparty@1.8.6*, we can associate this vulnerability with *copyparty* version *1.8.6*. Finally, once the dependencies have been attributed, two dumps have been extracted, one from our vulnerability database to be loaded into MongoDB, and the other from the attributed graph to be loaded into Neo4J.^6^ All the tasks shown in Fig. 3 have been implemented using Python version 3.10 and are included as an extension of **Depex** tool [2]; and the corresponding libraries for HTTP connections, PostgreSQL, Mongo and Neo4J database connections, and csv file creation.

## 5 Dataset Characterisation

This section provides a descriptive overview of selected characteristics of the dataset, focussing on versioning and vulnerability metrics in the four package managers (NPM, PyPI, RubyGems and Cargo). These statistics aim to help better understand the structure of the data set and the type of information it contains at that point in time.^7^ They may be useful for researchers interested in replication studies, dependency risk modelling, or large-scale software ecosystem analysis.

The remainder of this section is organised into three parts. First, Subsection 1 reports the number and proportion of the latest vulnerable versions across ecosystems, providing a snapshot of how vulnerabilities persist in current releases. Subsection 2 focusses on the distribution of vulnerabilities by severity level and the associated number of known exploits, providing information on potential impact and exploitation patterns. Finally, Subsection 3 examines how frequently packages depend on vulnerable components, revealing the extent to which transitive dependencies contribute to ecosystem-wide exposure. Together, these metrics describe key dimensions of the dataset and can inform future research on software supply chain security.

1. **Overview of vulnerable latest versions**

This subsection provides aggregated data about the number of vulnerable package versions, with a specific focus on the latest available versions. This information helps to assess whether vulnerabilities persist in recent releases or are mostly confined to outdated versions. Table 3 summarises the number of total versions, vulnerable versions, and vulnerable *latest* versions for each package manager. For PyPI, 329 of the 78,476 vulnerable versions are the latest vulnerable versions (approximately 0.42 %). RubyGems includes 919 vulnerable latest versions among its 23,491 vulnerable versions (3.91 %). Cargo has the lowest count, with 53 vulnerable latest versions among 707 (7.5 %). NPM presents the largest amount, with 13,557 vulnerable latest versions out of 195,515 vulnerable versions (6.93 %).

**Table 3:** Number of vulnerable versions for each package manager.

Manager	N° of Versions	N° of Vulnerable Versions	N° of Vulnerable Latest Versions
NPM	50,943,372	195,515	13,557
PyPI	6875,330	78,476	329
RubyGems	1738,773	23,491	919
Cargo	1393,371	707	53
Total	60,950,846	283,592	14,858

Overall, the dataset contains 14,858 vulnerable latest versions, representing 5.24 % of the 283,592 vulnerable versions identified across all package managers. This enables users to analyse the persistence of vulnerabilities in actively maintained packages or to select subsets of packages with up-to-date yet vulnerable releases. In terms of general vulnerability rates, NPM shows 0.38 % (195,515 out of 50,943,372 versions), PyPI 1.14 % (78,476 out of 6875,330), RubyGems 1.35 % (23,491 out of 1738,773), and Cargo 0.05 % (707 out of 1393,371). These numbers can assist in ecosystem-level comparisons and may serve as reference points for assessing the relative density of vulnerabilities.

Equivalent packages across ecosystems may report different numbers of known vulnerabilities. As shown in Table 4, gRPC, maintained by Google, appears with 261 vulnerabilities in NPM, 785 in PyPI, and 3571 in RubyGems. Similarly, Protocol Buffers reports 156 vulnerabilities in NPM, 280 in PyPI, 2365 in RubyGems, and 63 in Cargo. The discrepancies can also be seen with Apache Arrow and WebAssembly, whose vulnerability counts differ widely between ecosystems. This may be due to differences in naming conventions, vulnerability disclosure processes, or the propagation of vulnerabilities between upstream and downstream dependencies. Understanding these variations can support future research on cross-ecosystem alignment and divergence.

**Table 4:** Number of vulnerabilities of packages in different managers.

Owner	Tool Name	NPM	PyPI	RubyGems	Cargo
Google	Protocol Buffers	156	280	2365	63
Google	gRPC	261	785	3571	0
Apache	Apache Arrow	0	90	0	0
Bytecode Alliance	WebAssembly	0	174	16	0

2. **Distribution of vulnerabilities by impact**

This subsection provides a breakdown of vulnerabilities by severity level and their associated exploit counts. This allows users to assess how vulnerabilities of different impact levels are represented in the dataset and how frequently they have been exploited in the wild. Table 5 details the number of vulnerabilities and exploits recorded, grouped by impact level: Low, Medium, High, and Critical. This classification follows standard severity labels commonly used in security databases and enables users to assess the risk profile of the dataset.

**Table 5:** Number of vulnerabilities and exploits by impact.

Impact	N° of Vulnerabilities	N° of Exploits
Low	2688	30
Medium	49,847	1161
High	45,280	1538
Critical	12,980	773

Low-impact vulnerabilities are the least common (2688) and are associated with 30 exploits. Medium-impact vulnerabilities account for 49,847 cases, with 1161 recorded exploits. High-impact vulnerabilities are also prominent (45,280), with 1538 associated exploits. The dataset includes 12,980 Critical vulnerabilities, which have 773 recorded exploits. Although considered less severe, low and medium impact vulnerabilities may still be relevant for studies on exploit frequency, attacker preferences, or long-tail risk. These metrics offer insight into how the severity of the impact correlates with the exploitation rates in the dataset. Users may use this information to filter or prioritise subsets of vulnerabilities according to their

3. **Prevalence of indirect dependencies on vulnerable packages**

This subsection focusses on the number of package versions that depend, directly or transitively, on at least one vulnerable package. Such dependencies can propagate risks even if the dependent package itself does not contain a vulnerability. Table 6 presents the number and proportion of versions that depend on at least one vulnerable package. For PyPI, 1325,642 versions (20.24 %) require vulnerable packages. RubyGems has 1089,360 such versions (62.64 %), and Cargo 265,672 (19.07 %). NPM, newly added to the dataset, exhibits 20,208,968 such versions, which represents 39.66 % of its total. These values reflect the potential exposure to vulnerabilities via transitive dependencies, which are often harder to track and mitigate. This part of the dataset may be useful for dependency management studies, package risk assessment, or supply chain security modelling.

**Table 6:** Number of versions requiring vulnerable packages for each package manager.

Manager	N° of Versions	Requiring Vulnerable Packages
NPM	50,943,372	20,208,968
PyPI	6875,330	1325,642
RubyGems	1738,773	1089,360
Cargo	1393,371	265,672
Total	60,950,846	22,889,642

This summary of metrics is intended to guide data users in selecting relevant subsets or in understanding potential dimensions of analysis. Conclusions are not drawn on causality, effectiveness of security practices, or risk management policies. By providing these aggregated statistics, the dataset becomes easier to navigate and more accessible to researchers with different backgrounds or goals.

## Limitations

Considering threats to validity is essential to ensure the quality of research. Wohlin et al. [17] classify these threats into four categories:

• **Internal validity:** This refers to factors that can unknowingly influence results. In our study, a key threat is the selection of package managers. We chose NPM, PyPI, Cargo, and RubyGems due to feasible extraction times (32, 20, 4, and 3 days, respectively), while others like Maven Central were excluded due to the complexity of data scraping. This decision may limit the generalisability of our findings.
• **External validity:** These threats affect the applicability of results to broader contexts. A major concern is **Temporal Validity**, as new packages and vulnerabilities emerge daily, requiring frequent update of the dataset. Furthermore, focussing on only three package managers poses a **Population Validity** threat, limiting generalisation to ecosystems such as NPM or Maven Central.

## Ethics Statement

The authors confirm that they have adhered to the ethical guidelines required for publication in Data in Brief. This study does not involve human participants, animal testing, or data obtained from social media platforms. Furthermore, the authors affirm that the work presented is original, has not been submitted elsewhere for publication, either in full or in part, and has received approval from all listed authors.

## Credit Author Statement

**A. Germán Márquez:** Visualisation, Conceptualisation, Methodology, Software, Data curation, Formal analysis, Writing - Original draft preparation. **Ángel Jesús Varela-Vaca:** Visualisation, Conceptualisation, Methodology, Supervision, Validation, Writing - Reviewing and Editing. **María Teresa Gómez López:** Visualisation, Conceptualisation, Methodology, Supervision, Validation, Writing - Reviewing and Editing.

## Data Availability

ZenodoData in Brief Material for Experimental Reproducibility (Original data).

## Footnotes

1 purl: https://github.com/package-url/purl-spec.

2 NPM JSON Replicate API: https://replicate.npmjs.com.

3 PyPI JSON API: https://docs.pypi.org/api/json/.

4 RubyGems dump website: https://rubygems.org/pages/data?locale=en.

5 Cargo dump website: https://crates.io/data-access.

6 The CSV file or dumps can be updated to incorporate new data as new versions or packages are released by package managers and new vulnerabilities arise.

7 Currently 15 May 2025.
